# Learning real-life cognitive abilities in a novel 360°-virtual reality supermarket: a neuropsychological study of healthy participants and patients with epilepsy

**DOI:** 10.1186/1743-0003-10-42

**Published:** 2013-04-23

**Authors:** Philip Grewe, Agnes Kohsik, David Flentge, Eugen Dyck, Mario Botsch, York Winter, Hans J Markowitsch, Christian G Bien, Martina Piefke

**Affiliations:** 1 Physiological Psychology, Bielefeld University, P.O. Box 10 01 31, Bielefeld, D-33501, Germany; 2 CITmed Project, Center of Excellence Cognitive Interaction Technology (CITEC), Bielefeld University, P.O. Box 10 01 31, Bielefeld, D-33501, Germany; 3 Computer Graphics and Geometry Processing, Faculty of Technology, Bielefeld University, P.O. Box 10 01 31, Bielefeld, D-33501, Germany; 4 Cognitive Neurobiology, Neurocure, Cluster of Excellence, Humboldt University, Dorotheenstrasse 94, Berlin, D-10117, Germany; 5 Bethel Epilepsy Centre, Mara Hospital, Maraweg 17-21, Bielefeld, D-33617, Germany; 6 Department of Psychology and Psychotherapy, Neurobiology and Genetics of Behavior, Witten Herdecke University, Alfred-Herrhausen-Straße 50, Witten, D-58448, Germany

## Abstract

**Background:**

To increase the ecological validity of neuropsychological instruments the use of virtual reality (VR) applications can be considered as an effective tool in the field of cognitive neurorehabilitation. Despite the growing use of VR programs, only few studies have considered the application of everyday activities like shopping or travelling in VR training devices.

**Methods:**

We developed a novel 360°- VR supermarket, which is displayed on a circular arrangement of 8 touch-screens – the “OctaVis”. In this setting, healthy human adults had to memorize an auditorily presented shopping list (list A) and subsequently buy all remembered products of this list in the VR supermarket. This procedure was accomplished on three consecutive days. On day four, a new shopping list (list B) was introduced and participants had to memorize and buy only products of this list. On day five, participants had to buy all remembered items of list A again, but without new presentation of list A. Additionally, we obtained measures of participants’ presence, immersion and figural-spatial memory abilities. We also tested a sample of patients with focal epilepsy with an extended version of our shopping task, which consisted of eight days of training.

**Results:**

We observed a comprehensive and stable effect of learning for the number of correct products, the required time for shopping, and the length of movement trajectories in the VR supermarket in the course of the training program. Task performance was significantly correlated with participants’ figural-spatial memory abilities and subjective level of immersion into the VR.

**Conclusions:**

Learning effects in our paradigm extend beyond mere verbal learning of the shopping list as the data show evidence for multi-layered learning (at least visual-spatial, strategic, and verbal) on concordant measures. Importantly, learning also correlated with measures of figural-spatial memory and the degree of immersion into the VR. We propose that cognitive training with the VR supermarket program in the OctaVis will be efficient for the assessment and training of real-life cognitive abilities in healthy subjects and patients with epilepsy. It is most likely that our findings will also apply for patients with cognitive disabilities resulting from other neurological and psychiatric syndromes.

## Background

The lack of ecological instruments in the field of clinical neuropsychology has been criticized frequently [1-4]. This criticism is particularly relevant in the field of complex higher cognitive functions such as (autobiographical) episodic memory [5-7] and executive functions [8]. Among a number of approaches to increase ecological validity [9], the use of Virtual Reality (VR) has been increasingly considered to allow for an ecologically valid assessment of everyday cognitive functions [10,11]. During the last two decades, a growing number of studies in the field of clinical neuropsychology used VRs for assessment and intervention purposes [for reviews see [12,13]. The VR technique allows for both control of experimental manipulations (e.g., different levels of complexity) and precise measures of subjects’ responses [14] within relatively natural, immersive settings [15,16].

As cognitive functions are known to be altered by age [17], there is a considerable need for rehabilitation programs in our aging society. In rehabilitation settings, shopping can be considered as one of the most important activities to maintain and/or regain elderly people’s and neurological and psychiatric patients’ independent everyday life functioning [18-20]. In spite of their high relevance to successful rehabilitation for neurological and psychiatric patients, only few of these everyday activities, including shopping, have been implemented in computer-assisted training programs and VR applications [21]. In an early report, Cromby et al. [20] investigated real life transfer of cognitive tasks learned in a virtual supermarket or other VR scenarios in students with learning difficulties using a two-week training intervention. In the experimental group, participants were presented with a shopping task in a virtual supermarket, while the control group was trained inside various VRs (e.g., a virtual house, a virtual city). The authors reported that the experimental group needed less time and bought more correct items in a subsequent shopping task in a real supermarket in comparison to the control group. Lee et al. [22] conducted a five day training in a virtual supermarket presented via a head-mounted display. On a descriptive statistical level, they could show effects of learning during the course of the five day learning program, but they did not report any inferential statistics. Rand, Weiss and Katz [23] developed a virtual version of the Multi Errands Test [MET; [24], a standardized test to assess and train multitasking behavior in a real shopping mall, by adapting the MET into a virtual mall presented via a video capture system. In the MET, the participant has to run several different given errands, which require a strategic planning because of restrictions and rules to be followed (e.g., order of the errands, time restrictions, different opening hours). Using this VR-MET, the authors investigated effects of ten training sessions in four stroke patients. They observed a decrease of rule breaks and non-efficient strategies in both the traditional and the virtual MET. Klinger et al. developed a virtual supermarket task presented on a 17” LCD screen to assess executive functions in different patient groups. Action planning was measured during a shopping task, which required the participants to plan his/her shopping according to a given shopping list [25]. Using this paradigm, the authors successfully showed in a number of studies impaired and spared aspects of executive functions in patients with mild cognitive impairment [25], stroke [26], Parkinson’s disease [27] and schizophrenia [28]. However, they did not report effects of training using their shopping task.

Available data demonstrate that VR supermarkets can efficiently be applied for the assessment and training of cognitive functions. Previous studies applied virtual supermarkets with relatively low resemblance to real life supermarkets [20] and the technical presentation was rather simple (e.g., use of small LCD-screens). Also, previous work has mainly focused on case study designs and descriptive statistics. Most importantly, VR supermarkets were used only for neuropsychological assessment, but not for training purposes.

Based on the current state-of-the-art of VR applications in neuropsychological diagnosis and rehabilitation, we developed a novel 360°- virtual reality supermarket displayed on a circular arrangement of 8 touch-screens – the “OctaVis” [29]. This 360°- apparatus allows for intuitive real world-like movements, as the participant is able to turn around and rotate freely and interact with virtual items by real world-like movements (e.g., reaching out with the arm and hand for articles). It offers the opportunity to display high-resolution 3D-graphics at 360°- field of view, which can be understood as an advantage over the aforementioned studies. The main aim of this study was to evaluate the efficacy of a new 360°- VR supermarket task for the ecologically valid assessment and training of cognitive functions. Therefore, we analyzed participants’ performance and level of presence in our new 360°- OctaVis, using a virtual shopping task. We propose that our technically more advanced presentation of the VR may induce stronger feelings of immersion and this will enhance the efficacy of training within the VR [30,31]. Our assumption is based on previous research showing positive effects of display size and participants’ field of view on the subjective feeling of immersion [32,33]. In particular, we hypothesize that training in our 360°-VR supermarket will lead to substantial learning in remembering and finding articles of a shopping list. Furthermore, learning of this list will not be interrupted by the introduction of a new, interfering shopping list. Finally, we assume participants’ level of immersion into the VR and feasibility of our task to predict levels of learning in our task. To address the issue whether training effects are comparable in a clinical sample, we also included a small sample of patients with focal epilepsy in our study. Due to the high incidence of memory impairments in this clinical group [34,35], patients were considered to have a special benefit from our VR training program of memory functions in an everyday-like context.

## Methods

### Participants

Participants were 19 healthy university students (5♂, 14♀). Mean age was 23 ± 3.45 (range 19 to 28 years). To ensure a homogeneous sample with average general cognitive functioning, we assessed participants’ IQ. Mean IQ was 109.9 ± 8.58. Participants’ medical history was assessed via a self-report questionnaire and a subsequent interview for detailed medical anamnesis. Participants with lifetime head injuries, severe medical illness, medication affecting the central nervous system, psychiatric diseases, and neurological diseases were excluded from the study. Also, participants with current consumption of illegal drugs or alcohol abuse were excluded.

We also examined an additional small sample of patients with focal epilepsy (*n* = 5). Mean age of patients was 35.04 ± 8.08 (range 25 to 47), mean IQ was 104 ± 9.23. Additional file 1 provides a detailed overview of the patients’ clinical and demographic data. In the clinical sample, neuropharmacological medication of epilepsy was not generally an exclusion criterion. However, patients who were treated with substances causing memory disturbances were excluded from the study.

Written informed consent was obtained from all subjects prior to participation, and the local ethics committee approved the study.

### Protocol

**Figure 1:**
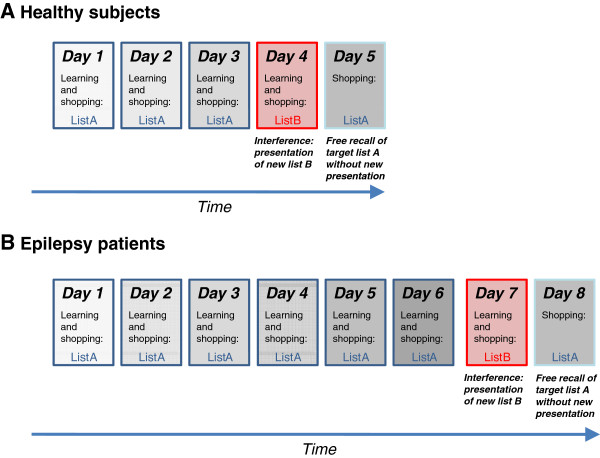
**Schematic overview of the VR training program used in the OctaVis. A**) During the first three days, healthy participants had to memorize and buy items of a target shopping list (list A), which was auditorily presented at the beginning of the trials. On day four, a new, interfering list (list B) was presented and participants had to memorize and buy only items of this interference list. On day five, participants had to recall and buy only items of the target list A, but without any further presentation and learning of list A. **B**) For the epilepsy patients, an extended program was used. This included six days of learning, on which the patients had to memorize and buy items of an auditorily presented target list (list A). On day 7, a new, interfering list (list B) was presented and participants had to memorize and buy only items of this interference list. On day 8, participants had to recall and buy only items of the target list A, but without any further presentation and learning of list A.

All healthy participants accomplished a five day training program in the VR (see Figure 1A). On day 1–3, subjects heard an auditorily verbal presentation of the same shopping list including 20 shopping items (list A). Thereafter, they were instructed to memorize and buy all items that had been presented. Participants walked through the supermarket and picked the learned shopping items out of the assortment of products of the VR supermarket. On day 1, before starting the VR shopping, participants accomplished a practice trial in a 3D room, which had the same size as the virtual supermarket, but did not include any articles and shelves. The main VR experiment was only started after participants had fulfilled the criterion of successful operation in this training room (i.e., walking straight, turning around, walking in curves) and reported to feel comfortable with the control system of the OctaVis. The required time for accomplishing the practice trial ranged from two to five minutes. Subjects were instructed to buy all items of the shopping list that they remembered as fast as possible, but without buying any products that had not been on the list. On days 2 and 3, list A was presented again to the participants, and the shopping task remained the same. On day 4, a different list was presented auditorily, which comprised 20 new shopping items (list B). List B was applied to induce interference into the training phase. Except for the novel shopping items, the task and the instructions remained the same. On day 5, participants were instructed to buy all items of list A which they remembered as fast as they could, without a new presentation of list A. Subjects were thus required to recall the shopping items of list A from the former learning trials on day 1 to 3. Moreover, subjects were told not to buy any items of list B. Start and end points in the supermarket were fixed. All participants started at the turnstile at the entrance of the VR supermarket on every trial. To finish the experiment, participants had to move to the end of the central cash point and tell the examiner that they had completed the task when they felt that they collected all items that they remembered. Navigation in the VR supermarket was not restricted besides real-life restrictions such as collision with shelves and walls of the supermarket. Participants could freely navigate through the VR supermarket, and there was no restriction of time to accomplish the task. Prior to the experiment, participants were told that they have to accomplish a shopping task, but they were not informed about details of the study design (e.g., target list, interference list, number of shopping products on each list). Moreover, they were informed about the daily and total duration of the experiment. While shopping, participants could not see the products that they had already bought. Both shopping lists included the same four different semantic categories: “beverages”, “hygiene items”, “groceries” and “households goods” with, each category containing five articles.

For the epilepsy sample, we administered an extended eight-day version of our supermarket training program instead of the five-day training applied in healthy adults. This modification was implemented since even healthy volunteers were unable to remember and buy all 20 items of the target list on day 3, that is, the day before the interference list was presented. The absence of ceiling effects in normal subjects indicates a rather high difficulty of our VR shopping paradigm, which is sensitive for the differentiation between high and low performers in healthy young adults. It is therefore likely that a three-day learning of the target list may be too short for learning both target list items and orientation in the VR supermarket in a clinical sample. The extended 8-day training thus included 6 days of training of the target list (instead of 3 days) before the interference list was presented. Except the prolonged learning phase of the target list, the study design was the same as in the five- day program. Figure 1B shows a schematic overview of the extended training program applied to patients with epilepsy.

### VR apparatus

**Figure 2:**
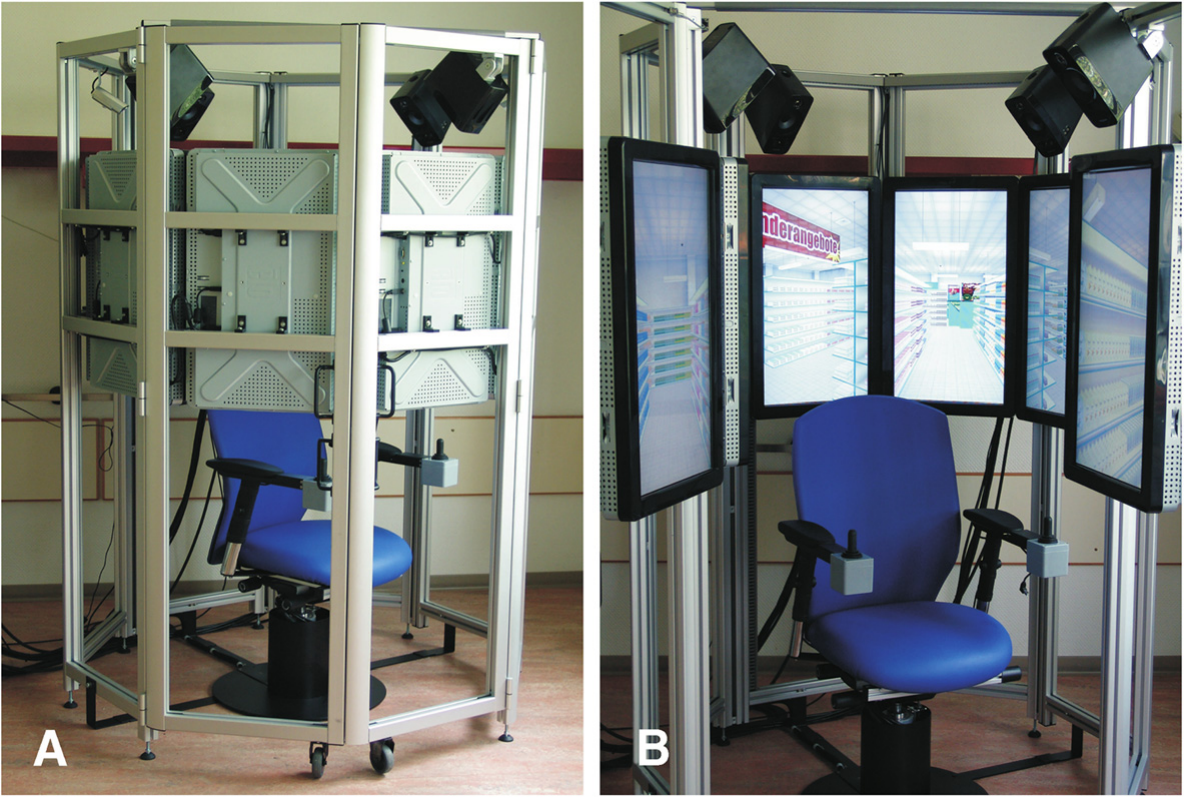
**Structure of the OctaVis, in closed (A) and opened (B) state.** The swivel chair is surrounded by a ring of eight LCD- touchscreens. Four speakers on top of the frame are used to provide auditory stimuli.

**Figure 3:**
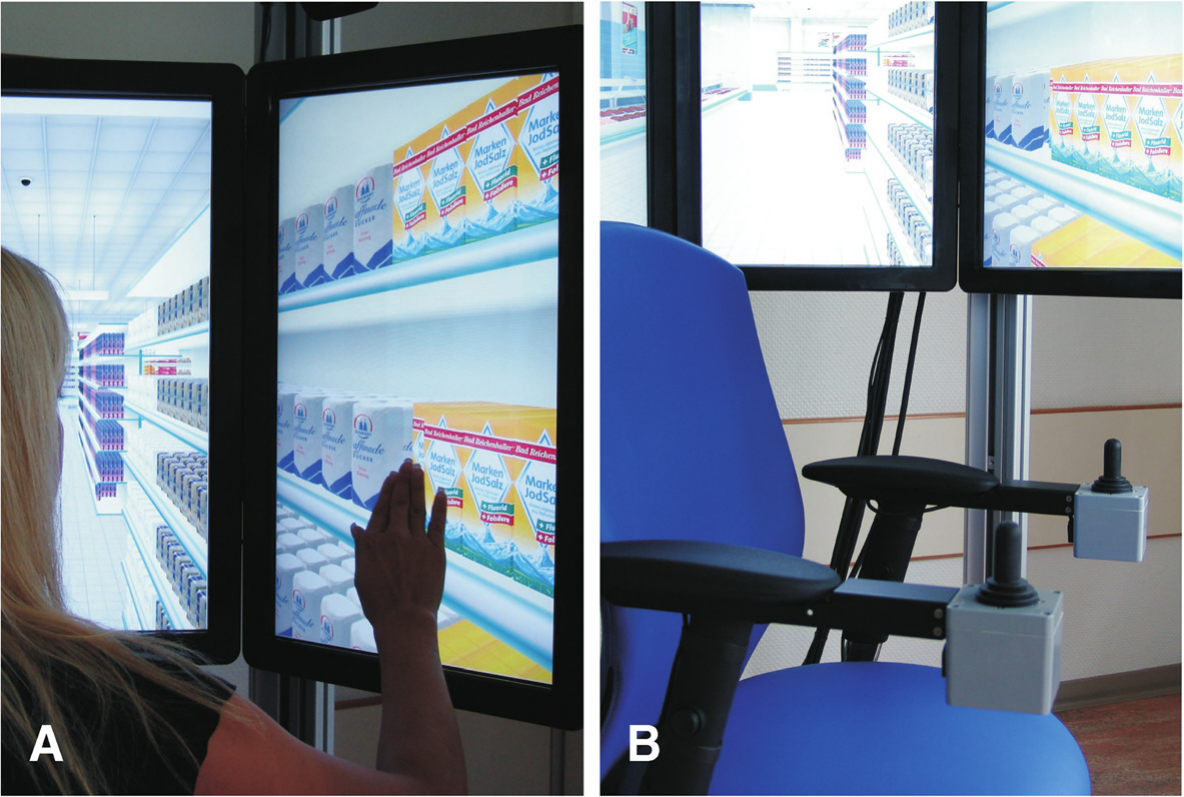
**Detail view of the LCD-touchscreens (A) and the swivel chair (B) of the OctaVis.** Items in the VR can be bought by tapping them on the screens. The items disappear from the screens after selection (**A**). Navigation in the VR is accomplished by chair rotation and the “throttle” joysticks for linear movement (**B**).

The VR was presented on a new 360°- VR apparatus, the “OctaVis“ [29]. The OctaVis consists of eight LCD-touch-screens, which are arranged in a circle around the participant (Figure 2A: OctaVis in a closed state; Figure 2B: OctaVis in an open state). Within this circle of eight screens the participant is sitting on a fixed swivel chair, which can be freely rotated. The orientation of the chair corresponds to the viewing and movement direction of the participant in the VR. Forward, backward and side movements are accomplished by using a “throttle joystick” (Metallux, Korb, Germany, http://www.metallux.de), which is installed on the chair’s arm-rest (Figure 3B). By tapping the LCD touch-screens, participants select the products they want to buy (Figure 3A). All products in the supermarket (i.e., list A and B, and all other distractor products) can be bought by tapping on it. As every product in the supermarket was displayed in an array with multiple units of the same category, only the single product that is bought disappears from the screens, but the remaining items of the same category remain visible.

### Virtual Environment

**Figure 4:**
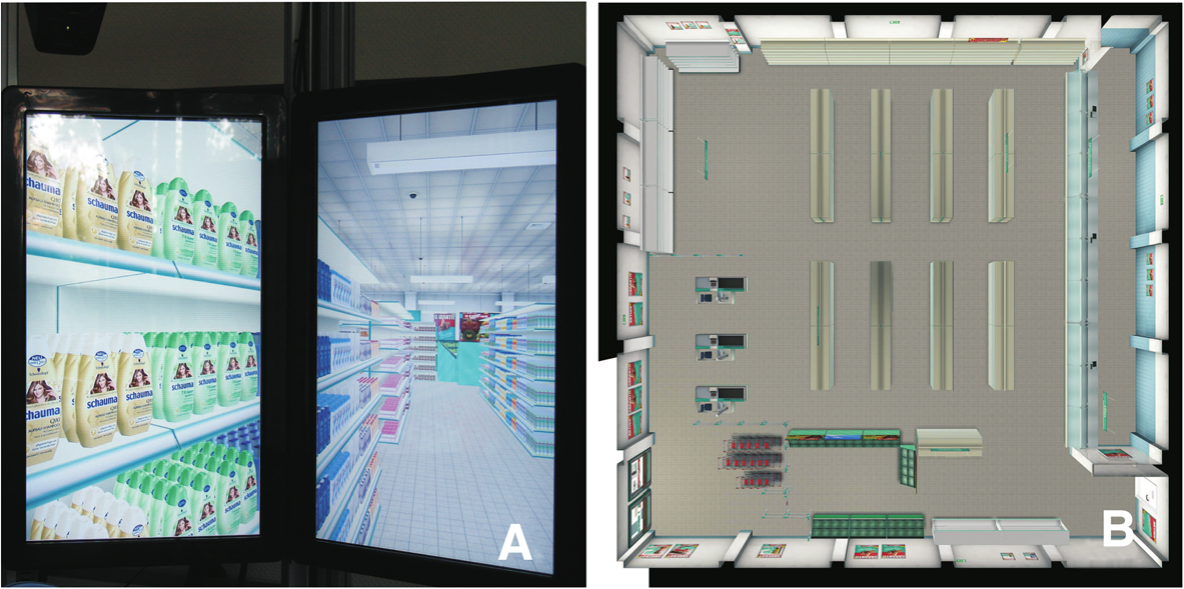
**Layout and design of the virtual supermarket displayed on the OctaVis.** Two out of eight LCD-touchscreens providing a detail view of the virtual supermarket with three shelves and one aisle (**A**). The top view shows the complete layout of the supermarket from the bird’s eye view (**B**). Note, that participants did not see the supermarket’s top view as shown in (**B**).

We used a virtual medium-sized supermarket, which had a structure comparable to that of a real supermarket (Figure 4). The VR supermarket was modeled according to a real standard supermarket in Germany with a size of 25 × 25 meters. All goods in the supermarket were designed referring to real brands and packages from common products that can be found in German supermarkets. The VR supermarket included a total of 73 types of products (i.e., comparable products of different brands; 20 items of list A + 20 items of list B + 33 filler items). These 73 types of products were available in different subtypes or brands (e.g., the product “tea” was available as “green tea”, “black tea”, etc.), which resulted in a total of 243 different products (each belonging to one of the aforementioned 73 types of products; see Figures 3 and 4A). All products were available in multiple quantities (e.g., 72 units of green tea) such that the VR supermarket contained a total of 51.764 selectable items.

None of the articles following directly one after the other in lists A or B were placed next to each other in the virtual supermarket. For acoustic stimulation, we used a low background sound, which before had been recorded in a real supermarket and consisted of sounds, which can be typically found in a supermarket (e.g., customers passing by, announcements, customers’ conversations). Sound files were provided via four stereo speakers, which were installed on top of the OctaVis (Genelec, Iisalmi, Finland, http://www.genelec.com, Figure 2). There were no virtual customers or other persons in the supermarket.

### Presence and immersion

As the emergence of feelings of presence can be considered as a result of individual characteristics of a person and/or properties of a particular virtual environment [31], we obtained two different measures of immersion:

To investigate the participants’ perceived level of presence during the shopping situation in the virtual supermarket (*state measure of immersion*), we applied the Presence Questionnaire [PQ; [36] after the first day of training (day 1) and, again, after the last day of training (day 5). The PQ is a self rating questionnaire that assesses participants’ momentary level of presence, or the feeling of being immersed into a particular VR. Participants’ answers can be given on a 7-point Likert-scale. It is constructed to assess a multi-faceted construct of presence. In a principal components factor analysis, Witmer et al. [36] revealed that four PQ-subscales account best for the total variance data. These are “involvement”, “sensory fidelity”, “adaptation/immersion”, and “interface quality”.

In addition, before beginning the VR training, participants completed the Immersive Tendencies Questionnaire [ITQ; [31]. The ITQ is a self-rating questionnaire assessing an individual’s general tendency to get immersed into a virtual environment (*trait measure of immersion*). Trait measures of immersion can be highly different between individuals. In contrast to the PQ, the ITQ thus does not measure a current state of presence at a particular point in time. Therefore we applied the ITQ only at one time point. The items of the ITQ each comprise a 7-point Likert-scale and comprise three subscales: “involvement”, “focus” and “games”.

To control for the occurrence of cybersickness, we also asked the participants whether they experienced symptoms of vertigo as an indicator of feasible cybersickness.

We also examined usability of both the task and the technical apparatus using a self-constructed explorative questionnaire, which included four items belonging to the “task”-subscale and four items belonging to the “technique”-subscale (Additional file 2). The questionnaire was designed in style of the PQ and was given to the participants directly after the PQ on day 1 and day 5.

Finally, we assessed participants’ figural memory with the Rey–Osterrieth Figure [ROF; [37] before the beginning of our program: Participants first had to copy a complex geometric figure (measuring visuo-constructive and planning abilities). Then, three and 30 minutes later, they were asked to freely recall this figure by drawing it again from memory.

### Measures

For behavioral measures of performance in the virtual supermarket, we considered the time required by the participants to buy all the shopping items they remembered (“time”) as well as the number of correctly picked items from the respective list (“correct products”) the adjusted number of correctly picked items from the respective list (“product-score”; i.e., number of correct items minus false positives minus repetitions) and the length of movement trajectories (“LMT”). The LMT is given in meters and refers to the distance travelled in the VR supermarket by each participant to perform the task on each day. LMT accordingly represents the length of movement trajectories the participant would have traveled in a real 25 × 25 m supermarket.

Since we mainly focused on the analysis of performance of a healthy control group in our novel VR training program, we were particularly interested in behavioral raw measures on each single day of training. Our approach of data analysis allowed us to assess in detail how performance changed between two single trials/days and on which stages of training significant changes of performance occurred. For the same reason, we were also interested in correlations between performance on single trials/days in our VR program and scores on standard neuropsychological tests of figural memory as well as measures of immersion.

### Statistical analyses

All statistical analyses were conducted using SPSS 17 (SPSS Inc., Chicago, Illinois). The general significance level was set to α = .05. To check for the assumption of normally distributed variables, we used the Kolmogorov-Smirnov-Test (α = .20). Associations between the measures of learning and the results of the applied questionnaires were calculated by using Pearson’s correlation coefficient, *r.* Effects of learning in the course of the training were analyzed using matched pairs t-tests in the case of a comparison between two single days. The comparison between days 3 and 5 was calculated to test for potential interfering effects of the distraction list B on free recall of the target list A. In cases where comparisons across more than two days were calculated, we used repeated measures ANOVAs. If the repeated measures ANOVAs revealed significant differences between trials, post-hoc comparisons for the differences between each single pair of days were conducted using dependent t-tests. Multiple post-hoc-comparisons were corrected using Bonferroni adjustment. The Bonferroni adjusted significance level for the post-hoc-comparisons was α = .0083.

Due to the small sample size (*n* = 5), nonparametric tests were chosen for the analyses including the clinical group. Thus, associations between two variables were calculated using Spearman’s rho, *ρ*_*s*_. Comparisons between single trials were analyzed using Wilcoxon signed rank tests.

## Results

The Kolmogorov-Smirnov-Tests revealed non- significant results for all variables used in our analysis, thus indicating normal distribution of the variables.

### Efforts of learning

**Figure 5:**
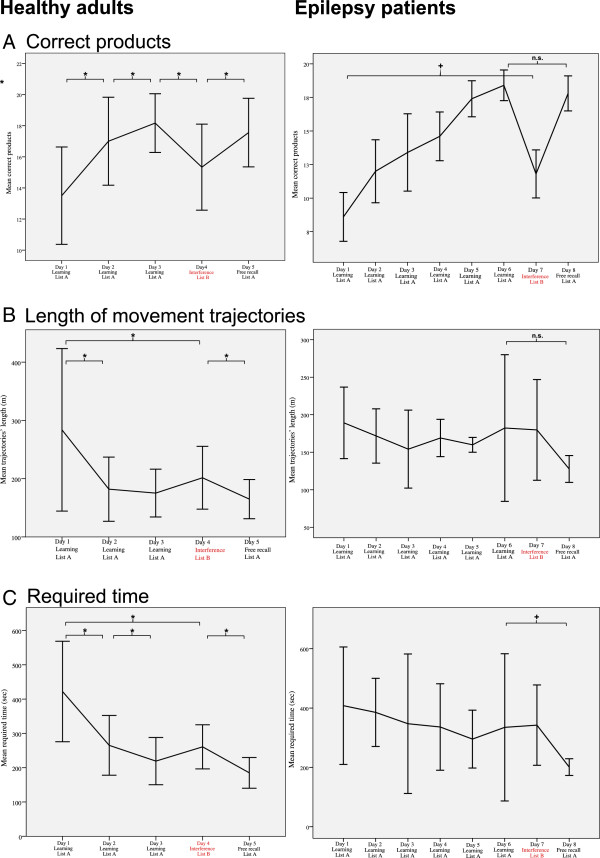
**Different measures of performance in the VR task for the healthy subjects (left side of the panel) and the epilepsy patients (right side of the panel. A**) The mean number of correctly bought products in the VR supermarket is shown for the five day program (left side) and for the eight day program (right side). **B**) The mean length of movement trajectories given in meters is shown for the five day program (left side) and for the eight day program (right side). **C**) The mean required time to buy all remembered products in the VR supermarket given in seconds is shown for the five day program (left side) and for the eight day program (right side). Error bars depict +/− 1 SD; “*” = significant difference between two days at *p* ≤ .0083 (corrected for multiple comparisons); “+” = significant difference between two days using the non-parametric Wilcoxon signed rank test to test the main hypotheses for the clinical group, *p* ≤ .05; “n.s.” = non- significant comparison.

There was an overall effect of learning over the course of the first three days of learning for the correct products (*WL* = .191, *F* = 33.81, *p* < .001, *η*^*2*^ = .809; Figure 5A, left side) and the product-score (*WL =* .177, *F* = 37.29, *p* < .001, *η*^*2*^ = .823; Additional file 3). Furthermore, there was an overall effect of learning considering all five days of training including the distractive trial on day 4 for the correct products (*WL* = .157, *F* = 18.82, *p* < .001, *η*^*2*^ = .843; Figure 5A, left side) and the product-score (*WL* = .164, *F* = 17.9, *p* < .001, *η*^*2*^ = .836). Comparing participants’ effort between two single days, there was a difference for the product-score from one day to the next for all five days (adjusted α < .0083). For the correct items, there was a difference from one day to the next for all five days (adjusted α < .0083; Table 1).

**Table 1:** Descriptive and inferential statistics of the correct products

		**Comparison**			
trial	mean (SD)	pair	*T*	df	*p*
day 1	13.50 (3.13)	day 1 vs. 2	4.48	17	< .001*
day 2	17.00 (2.82)	day 2 vs. 3	3.58	17	.002*
day 3	18.17 (1.89)	day 3 vs. 4	−3.82	17	.001*
day 4	15.33 (2.77)	day 4 vs. 5	3.01	17	.008*
day 5	17.56 (2.20)	day 1 vs. 4	2.02	17	.060
		day 3 vs. 5	−1.45	17	.165

SD = Standard deviation; df = degrees of freedom; * = significant difference at *p ≤* .0083 (adjusted for multiple comparisons).

The participants’ LMT decreased over the course of the three days of learning (*WL =* .614, *F =* 5.03, *p =* .02, *η*^*2*^ = .386; Figure 5B, left side). This effect of learning was also observed considering all five days of training including the distractive trial on day of our five day program (*WL = .*506, *F =* 3.42, *p =* .038, *η*^*2*^ = .494; Figure 5B, left side). Particularly, there was a difference in the required LMT for the single runs between day 1 vs. day 2, day 4 vs. day 5 and day 1 vs. day 4 (adjusted α < .0083; Table 2).

**Table 2:** Descriptive and inferential statistics of the length of movement trajectories

		**Comparisons**			
trial	mean (SD)	pair	*T*	df	*p*
day 1	283.72 (139.49)	day 1 vs. 2	−3.10	17	.006*
day 2	181.91 (55.06)	day 2 vs. 3	-.69	17	.496
day 3	175.19 (41.04)	day 3 vs. 4	2.25	17	.038
day 4	201.5 (53.92)	day 4 vs. 5	−3.31	17	.004*
day 5	164.83 (33.57)	day 1 vs. 4	−3.21	17	.005*
		day 3 vs. 5	−1.41	17	.178

Descriptive values are expressed in meters; SD = Standard deviation; df = degrees of freedom; * = significant difference at *p ≤ .*0083 (adjusted for multiple comparisons).

The required time for the single runs decreased over the course of the three days of learning (*WL =* .312, *F =* 17.6, *p <* .001, *η*^*2*^ = .688; Figure 5C, left side). This effect of learning was also observed considering all five days of training including the distractive trial on day 4 (*WL =* .23, *F =* 11.71, *p <* .001, *η*^*2*^ = .77; Figure 5C, left side). Particularly, there was a difference in the required time for the single runs between day 1 vs. day 2, day 2 vs. day 3, day 4 vs. day 5 and day 1 vs. day 4 (adjusted α < .0083; Table 3).

**Table 3:** Descriptive and inferential statistics of the required time

		**Comparison**			
trial	mean (SD)	pair	*T*	df	*p*
day 1	422.06 (146.5)	day 1 vs. 2	−4.76	17	< .001*
day 2	265.06 (87.12)	day 2 vs. 3	−3.84	17	.001*
day 3	219.22 (68.83)	day 3 vs. 4	2.80	17	.012
day 4	260.72 (64.26)	day 4 vs. 5	−5.45	17	< .001*
day 5	184.94 (44.72)	day 1 vs. 4	−6.03	17	< .001*
		day 3 vs. 5	−2.79	17	.012

Descriptive values are expressed in seconds; SD = Standard deviation; df = degrees of freedom; * = significance of difference at *p ≤ .*0083 (adjusted for multiple comparisons).

### Immersion and presence

Neither the total score of the ITQ nor one of the ITQ- subscales was correlated with the number of correct products, the product-score, the LMT or the required time.

The PQ total score increased from day 1 (*M* = 137.26, *SD* = 18.62) to day 5 (*M* = 143.89, *SD* = 13.47), but there was only a trend for a significant difference (*t* = −1.89, *p* = .075, *d* = .433). The PQ-subscale “immersion/adaptation” increased from day 1 (*M* = 40.74, *SD* = 6.62) to day 5 (*M* = 45.53, *SD* = 4.91; *t* = −2.76, *p* = .013, *d* = .632).

The PQ-subscale “immersion/adaptation” was correlated with the required time on day 3 (*r* = −.569, *p* = .014), the product score on day 2 (*r* = .473, *p* = .047) and day 3 (*r* = .546, *p* = .019) and the number of correct products on day 3 (*r* = .551, *p* = .018). The PQ-subscale “sensory fidelity” was correlated with the product-score on day 1 (*r* = −.539, *p* = .021). The PQ-subscale “involvement” was correlated with the LMT on day 1 (*r* = .564, *p* = .015) and day 4 (*r* = .623, *p* = .006). Table 4 summarizes the main results of the correlational analyses.

**Table 4:** Person correlations between VR performance and measures of immersion, figural memory and task feasibility

		**PQ Immersion**	**ROF 30’ recall**	**Feasibility**
Product-Score	Day 1	.258	*.*365	−0.83
	Day 2	.473*	.289	.048
	Day 3	.546*	.415	.059
	Day 4	-.188	.242	.178
	Day 5	.218	.527*	.249
Time	Day 1	-.097	-.109	-.357
	Day 2	-.404	-.055	.226
	Day 3	-.569*	-.262	-.041
	Day 4	-.210	-.066	-.200
	Day 5	-.173	.092	.044
LMT	Day 1	-.167	-.277	-.596*
	Day 2	-.054	-.074	-.091
	Day 3	-.167	-.355	-.321
	Day 4	.143	**-.414**	-.631*
	Day 5	.145	-.011	-.378

Values represent Pearson correlation coefficients; PQ Immersion = Presence Questionaire subscale “immersion/adaptation”; ROF 30’ = delayed recall of the Rey Oesterrieth Figure; Feasibility = questionnaire subscale “task”; LMT = Length of movement trajectories.

* = significant correlation at *p* ≤ .05.

### Figural memory

Participants’ mean T-Score in the immediate recall of the ROF after three minutes was 55.58 (*SD* = 10.43), the mean delayed recall after 30 minutes was 53.16 (*SD* =10.63).

**Figure 6:**
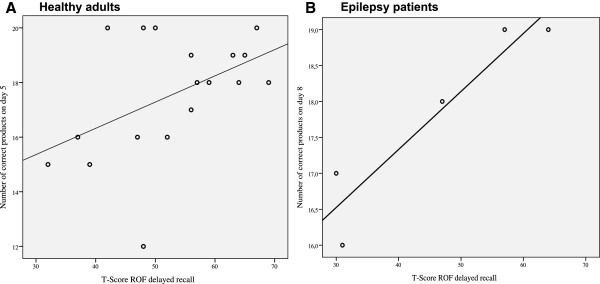
**Correlations between the delayed recall of the ROF and the number of correct products for the healthy adults (A) and the epilepsy patients (B).** The x- axis represents the delayed recall of the ROF after a 30’ delay and is given in T-Scores (*M* = 50, *SD* = 10). The y- axis represents the free recall of the target list on day 5 (**A**) and day 8 (**B**), respectively. ROF = Rey–Osterrieth Figure.

The immediate recall of the ROF was correlated with the product-score on day 5 (*r* = .484, *p* = .042). The delayed recall in the ROF was correlated with the product score on day 5 (*r* = .527, *p* = .024), as well the number of correct products on 5 (*r* = .472, *p* = .048; Figure 6A). Table 4 summarizes the main results of the correlational analyses.

### Feasibility questionnaire

The task-subscale of our explorative questionnaire significantly increased from day 1 (*M* = 17.53/28, *SD* = 2.39) to day 5 (*M* = 19.32/28, *SD* = 2.08; *t* = −4.04, *p* < .001, *d* = .927). The technique-subscale of our explorative questionnaire significantly increased from day 1 (*M* = 22.58/28, *SD* = 3.98) to day 5 (*M* = 27.74/28, *SD* = 3.02; *t* = −4.19, *p* < .001, *d* = .963).

The task-subscale was significantly correlated was the LMT on day 1 (*r* = −.596, *p* = .009) and day 4 (*r* = −.631, *p* = .005) and the number of correct products on day 1 (*r* = .476, *p* = .046). Table 4 summarizes the main results of the correlational analyses.

On day 1, nine of nineteen participants reported signs of vertigo, on day 5 only two of nineteen participants reported signs of vertigo. None of the participants cancelled participation in our study due to vertigo.

After the last training trial on day 5, participants were asked whether they used any kind of strategy to remember the items of the lists during the experiment via a self-constructed questionnaire. In particular, they were firstly asked whether they had applied any kind of strategy to remember the items of the shopping lists. If the application of any strategy was affirmed, participants had to describe and write down the applied strategy by their own words. Multiple answers were allowed. The following strategies were mentioned by the participants: “Dual-coding“ (i.e., the association of verbal information with visual-spatial information [design of each product and/or its localization in the VR supermarket]; 41%), “semantic clustering “(17%), “recognition” (6%), “verbal rehearsal” (6%), “serial clustering” (6%), “body-part method” (i.e., association of items with parts of the body; 6%), “counting of products” (6%), other (12%).

### Results of the clinical sample

Because of the small sample size, learning efforts across the eight day training program could not be calculated by repeated measurement ANOVAs. However, Figure 5 (right side) provides an explorative overview of the learning efforts (i.e., correct products, LMT, and time) of the clinical group. Concerning the main hypotheses, participants bought more items on day 7 (*median* = 12, *range* = 4) in comparison to day 1 (*median* = 9, *range* = 5; *Z* = 2.04, *p* = .042; Figure 5A, right side). The required time for shopping decreased from day 6 (*median* = 235, *range* = 575) to day 8 (*median* = 187, *range* = 64; *Z* = 2.02, *p* = .043; Figure 5C, right side). The LMT and the number of correct products did not differ between day 6 and day 8. There was a trend for a correlation between the number of correctly bought products on day 8 and the immediate (*ρ*_*s*_ = .872, *p* = .054) and the delayed recall (*ρ*_*s*_ = .872, *p* = .054; Figure 6B) of the ROF.

## Discussion

In this study, we investigated the feasibility of a VR supermarket application presented in the OctaVis, a novel 360°- VR apparatus, for the assessment and training of neuropsychological functions in a sample of healthy young adults and a small clinical sample of patients with focal epilepsy. Healthy participants learned a list of shopping articles and subsequently bought them in a virtual supermarket on three consecutive days. On day 4, participants learned and bought items of a new, distractive list. Finally, on day 5, participants had to buy only the items of the target list, but without a new presentation of this list. Results show increasing levels of learning throughout the task as well as high levels of subjects’ immersion in the VR. Moreover, performance in our task was significantly correlated with a measure of figural-spatial memory. The time needed for completion of the shopping task was significantly longer on day 1 than on day 4 (application of the distractive list B), indicating that visual-spatial familiarity with the structure of the supermarket decreased the immediate distracting effect of list B. Also, we did not observe a decrease of performance from day 3 to day 5, thus indicating that learning was not significantly interrupted by the introduction of a new interfering list. Importantly, immersion was positively correlated with the number of correctly bought products, suggesting that immersive feelings may enhance cognitive performance in everyday-like neuropsychological tasks.

In the course of training in the OctaVis, there was a considerable improvement of cognitive performance across training sessions. This effect of learning was observed for several concurrent measures: Participants successively needed less time and shorter movement paths to accomplish the task and bought a higher number of correct products, which was even valid when correcting for incorrect products and repetitions. Moreover, performance of the ROF was positively correlated with the number of correctly bought products. Finally, our main findings could be replicated in our clinical sample showing (a) comprehensive and stable learning, (b) no negative effect on performance after the introduction of the interference list, and (c) and association of measures of VR performance and the ROF in our patients with epilepsy.

We suppose that these comprehensive results of learning do not only reflect verbal learning as a consequence of the repeatedly presented shopping-list. Rather, improvement on the different but converging measures support the view that learning occurred mainly on verbal, visual-spatial, executive, and familiarity levels [20]. Although our data do not directly allow for conclusions on multi-layered learning, we propose that successful learning in the VR shopping task depends on the integration of at least verbal and visual-spatial modalities of learning, that is, a form of dual-coding. Moreover, the task requires executive abilities, in particular, visual-spatial planning strategies. This view is supported by the correlations between the ROF and learning scores in our VR task. The ROF represents visual-spatial memory functions, which are mostly independent of verbal abilities [38]. Besides visual-spatial memory abilities, the ROF also requires planning and structuring abilities and has correspondingly been used as a measure of planning and organization in previous studies [39-45]. We therefore propose that the correlation between our task and the ROF might represent the task’s requirement of at least verbal learning and non-verbal figural learning, as well as executive abilities, thus supporting our argument of multi-layered learning in the VR supermarket. Furthermore, our idea of a multi-layered learning process is also supported by the strategies participants used to accomplish the task. The use of dual coding, which implies the dual coupling of words of the shopping list with the visual representation of the respective product in the VR supermarket, was the memory strategy that was most frequently reported by our participants. It is most likely that the VR shopping task also makes demands on visual-spatial orientation and way-finding. However, our data do not contribute to this issue such that this assumption remains speculative. In our study, we did not aim at offering a full psychometric validation of our novel instrument. Furthermore, procedural learning and habituation to both technical control of the shopping task and the visual spatial structure of the VR environment may also have contributed to efficient learning. Eventually, our idea of a multi-layered learning that took place in our VR task goes in line with the concept of multiple memory systems that represent different subtypes of memory and learning processes [6,46,47].

Interestingly, the data show evidence for further learning from day 1 (first entering the supermarket) to day 4 (entering the familiar supermarket after having heard a new shopping list) for the time required for the shopping task and the LMT, but not for the product-score. This decrease of time and LMT on day 4 (relative to day 1) indicates that figural-spatial learning of the supermarket’s routes and layout occurred, which may be relatively independent of verbal memory of list A articles and their localization in the supermarket. This dissociation between verbal and figural-spatial learning further confirms our aforementioned idea of multi-layered learning. In particular, we suppose that participants created a cognitive map [48,49] of the virtual supermarket. Previous studies are in accordance with this assumption. The idea that generation of cognitive maps could be enhanced by learning in a VR [50], was supported by Tong et al. [51] who found that active performance in a VR-pathfinding task enhanced the accuracy in a subsequent drawing of a cognitive map of the landmarks in the VR. Results thus suggest that visual-spatial learning might have played a central role for task performance in our task besides verbal learning of the products. This assumption is in good accordance with the frequent application of dual-coding strategies reported by the volunteers (i.e., verbal and visual-spatial representation).

Insofar, our results are in line with a study of Brooks et al. [52], who found a dissociation between object and spatial learning in a VR. While the spatial layout of the VR could be recalled more accurately by participants who actively (vs. passively) navigated through the VR, the mere learning of objects placed in a VR was independent of (active vs. passive) navigation in the VR [52]. Moreover, in a study of patients with traumatic brain injury, Matheis et al. [53] found a dissociation between patients’ impaired list learning and spared visual memory performance.

We propose that learning of the layout of the VR supermarket took place on the first two days of training since the data show the highest increases of performance from day 1 to day 2. Moreover, the interference list did not affect performance in our VR shopping paradigm. Thus, there were no significant differences between all measures of performance on day 3 and day 5 (i.e., product-score, LMT, and time). Hence, our initial hypothesis that learning will not be interrupted by the introduction of a new shopping list was corroborated by the data, that is, shopping performance of the target list was comparable on days 3 and 5, although we inserted new learning materials in between these trials. It is therefore reasonable to assume that visual-spatial learning supported the emergence of a multi-layered representation of the shopping articles included in the target list. Importantly, we replicated this result in our preliminary study of the small clinical sample of patients with focal epilepsy. This argues in favor of a stable and robust representation of learned multi-layered information after an initial learning phase of the visual-spatial layout of the VR supermarket in both healthy controls and clinical samples.

For our present data, we suppose that interaction with the highly immersive 360°- VR OctaVis may have prompted multi-modal learning. There is considerable evidence from studies in children and adults that multi-modal learning is more efficient, deep and stable over time than unimodal learning [54-58]. Multi-modal learning may also enhance the feeling of presence and learning in VRs [59,60]. In addition, combined training of multiple cognitive functions could be shown to be an efficient strategy for rehabilitation of memory problems [61]. It is reasonable to assume that both the realistic layout of the supermarket and the real-life like interactive movements (e.g., turning around, reaching out with the arm and hand for an article) may have supported the integration of visual-spatial and motor learning, and therefore facilitated the formation of episodic in contrast to mere semantic memory contents [62], which makes our task a more precise measure of real-life cognitive performance. Most likely, immersion in the VR is a key player in the emergence of efficient multi-modal learning since it builds up the basis for real-life like integration of visual perception, motor-action and visual-spatial memory [52,63].

With regard to immersion, we observed increasing levels of subjective immersive feelings during the course of our training program in the 360°- VR supermarket. Insofar, our results are in good agreement with Lee et al. [22] who could also show participants’ increasing levels of subjective immersion into the VR in the course of a five day intervention. In our study, it is likely that participants became more and more familiar with the supermarket and the task and could thus successively immerse better into the VR during the course of the training intervention [64,65]. Increase of immersion may well be related to learning success. The correlations between measures of learning (i.e., product-score, time) and intensity of immersion, which both increased during the course of the task further supports this view. Our results provide first evidence that learning success depends at least in part on subjective immersive feelings. Eventually, our results are in accordance with the findings of previous studies showing that intense feelings of immersion may be associated with higher levels of task performance in a more general sense [31] and better treatment success in psychotherapy settings where VR is used to cure different forms of phobias [30].

As in previous VR studies using the PQ [22,66-68] we observed relatively high levels of immersion. On the one hand, this further underlines our apparatus’ technical feasibility for presenting highly immersive VR. We particularly assume that participants’ immersion was at least in parts enhanced by our novel apparatus with its specially designed 360°- view, the multi-sensory (i.e. visual, motor and auditory) design and the intuitive and interactive control. This is in line with several former research showing positive effects of field-of-view size [32,33], multi-modal integration [69] and active control on immersion [70]. On the other hand, it can be also supposed that the high levels of immersion found in our study will beneficially contribute to feasible transfer effects as immersion is thought to be a critical factor to enhance transfer from VR to real-life situations [71].

In contrast to these correlational associations between momentary presence and immersion measured with the PQ and task performance in the OctaVis, we did not find any association between performance in the OctaVis and the scales of the ITQ. Thus, a subject’s individual tendency for immersion was not found to be a critical factor for our VR training paradigm. We therefore conclude that performance in our paradigm mainly depends on the momentary level of immersion into the VR (as assessed with the PQ), rather than on a subject’s general personal trait or capability to get involved or immersed (as assessed with the ITQ). This aspect is of high importance for a future routine clinical application of VR scenarios in the OctaVis since the efficiency of training in the OctaVis should accordingly be independent of a person’s individual trait to get immersed into VR. This distinction allows for successful application of our program to a wider range of participants and does not restrict the application to participants with computer experience. Certainly, a further investigation examining a sample with a wider range of ages would add important information to this issue.

As it is important to use generally valid, “cross-media” measures of presence as well as specific measures that fit the proper and special technical features of the VR and its control devices [72], we also looked at the way participants subjectively got along with our particular task (represented by the task-subscale) and the technical apparatus (represented by the technique-subscale). We observed high scores in both subscales, which even increased significantly in the course of our program and were correlated with measures of learning in the VR task, thus indicating both an easy-to-handle control of our technical devices as a training program that seems intuitive and easy to handle. However, our questionnaire’s design was of explorative nature and scores may not be interpreted in terms of established measurements.

In summary, our findings provide preliminary evidence that our novel VR supermarket paradigm presented in the OctaVis may efficiently be applied for the assessment and training of real-life cognitive functions in healthy subjects and patients with focal epilepsy. However, we acknowledge some limitations and caveats interpreting our study. First, we did not control for each of the proposed levels of learning in particular. For example, we could not assess a verbal list learning paradigm as this would have interfered with the learning in our virtual paradigm. Although our study does not claim to offer a full-scale validation study, the application of additional specifically related “traditional” neuropsychological tests (e.g., of verbal memory) would presumably have added important information about single cognitive processes involved in performance in our novel VR paradigm. In particular, a labyrinth task needs to be included in future studies to assess the roles of visual-spatial orientation and way-finding in the VR shopping task. Moreover, list learning paradigms like the California Verbal Learning Task [73] or the Verbal Learning- und Memory Test [German adaptation of the Auditory Verbal Learning Test; [74], which comprise the most established measures of verbal memory [75], might offer important information on the role of verbal memory in the VR shopping task. Thus, the question whether both verbal and visual memory processes might have played a role could have been adressed more directly. In future experiments, we will hence offer additional measures of verbal memory performance. Second, our results should be replicated with a sample of older participants to evaluate generalization of our results to a group of participants with less computer experience. We did not directly address the participants’ motivation during shopping in the VR supermarket on each day of training. It is reasonable to assume that besides cognitive capacity, different levels of motivation and effort could have had an influence on the participants’ performance in the VR supermarket. Moreover, participants’ experience of cybersickness needs to be considered as another constraint of our study. Using a questionnaire on experiences of cybersickness we aimed at identifying sources of cybersickness during training in the OctaVis. Most participants reported a flicker in their peripheral field of view as an eliciting factor of nausea. This flicker is mainly technically related to fast movements in the VR, a relatively low frame-rate of the VR system (i.e. a low speed of re-generation of the VR environment), and the large field of view [76]. In our current studies of training in the OctaVis, we have accelerated the frame-rate and limited the maximum speed of movement inside the VR to reduce symptoms of cybersickness elicited by these factors. These changes of technical parameters resulted in a considerable reduction of reports of cybersickness. Finally, our task’s psychometric properties must be further evaluated. Therefore, we will apply the OctaVis to different populations to examine its validity in laboratory and further clinical settings. We currently elaborate different forms of feedback, which can be suitable for different patient groups. In parallel, we compare our VR program with already established training paradigms.

## Conclusions

Based on diverse concordant measures of visual-spatial, strategic, and verbal cognition, we showed evidence for a comprehensive and multi-layered learning success in the course of the training. Correlations between measures of multi-layered learning and scores on classical neuropsychological tests of visual-spatial cognition corroborate the view that our findings do not only depend on verbal learning of the shopping list. Moreover, we were able to demonstrate the feasibility of the VR supermarket paradigm presented in the OctaVis by high scores on both an established presence questionnaire and a self-constructed questionnaire, which was specifically related to our technical device. Importantly, we also provide strong evidence of a relation between the level of immersion and task performance. We conclude that we have developed and tested a novel 360°- VR environment, which demands real world-like visual-spatial and motor actions and thus allows for the training of the respective cognitive abilities. Importantly, results of our sample of patients with focal epilepsy corroborate the main findings of our basic healthy sample, thus giving preliminary evidence of a replication of comparable effects in a clinical sample. We propose that our technical device and neuropsychological paradigm can efficiently be used for assessment and training of real life-based cognitive functions. Future studies providing both additional validation data and clinical evidence are needed to corroborate the efficiency of cognitive training interventions in the OctaVis in patients with different neurological and psychiatric diseases.

## Competing interests

We disclose any actual or potential conflicts of interests including any financial, personal or other relationships with other people or organizations within three years of beginning the work submitted that could have inappropriately influenced our work.

## Authors’ contributions

PG and MP designed the study’s rationale and protocol and the manuscript draft. PG was involved in the study’s organization and statistical analyses of the data. DF helped in the preliminary data processing. AK and PG conducted the examinations and the experiments. MB, YW, ED, MP and DF developed the technical devices and the software. MP and MB are heads of the CITmed-project providing supervisory knowledge and help for the development and the conduction of the study. HJM is head of the Physiological Psychology-Unit providing help and advice for the manuscript draft and the study’s rationale. CGB is head of the Epilepsy Centre Bethel providing knowledge, help and advice with the clinical sample. All authors have read and approved the final manuscript.

## Supplementary Material

Additional file 1 Clinical and demographic characteristics of the epilepsy patients subgroup. Click here for file

Additional file 2 **Explorative usability questionnaire.** The self-constructed questionnaire includes eight items. The first four items comprise the task-score; the last four items comprise the technique-score. Items are translated from the German version used in the study. Click here for file

Additional file 3 **Mean product score for the single trials of the five day VR program.** Days 1 to 3 describe the product score (correct products minus false positives minus repetitions) for the consecutive learning of list A. Day 4 describes the product score for the interfering list B. Finally, day 5 describes the product score for the free recall of list A after the interference on day 4 (Note that on day 5, list A was not presented to the subjects again, but items should be recalled from the former learning trials). Error bars depict +/- 1 SD. Click here for file

## References

[B1] Spooner DM Pachana NA Ecological validity in neuropsychological assessment: a case for greater consideration in research with neurologically intact populations Arch Clin Neuropsychol 2006 21 327 337 10.1016/j.acn.2006.04.004 16769198

[B2] Chaytor N Schmitter-Edgecombe M The ecological validity of neuropsychological tests: a review of the literature on everyday cognitive skills Neuropsychol Rev 2003 13 181 197 15000225 10.1023/b:nerv.0000009483.91468.fb

[B3] Deisinger K Markowitsch HJ Die Wirksamkeit von Gedächtnistrainings in der Behandlung von Gedächtnisstörungen Psychol Rundsch 1991 42 55 65

[B4] Heinrichs RW Current and emergent applications of neuropsychological assessment: Problems of validity and utility Prof Psychol Res Pract 1990 21 171 176

[B5] Piefke M Weiss PH Zilles K Markowitsch HJ Fink GR Differential remoteness and emotional tone modulate the neural correlates of autobiographical memory Brain 2003 126 650 10.1093/brain/awg064 12566286

[B6] Markowitsch HJ Piefke M The functional neuroanatomy of learning and memory Handbook Clin Neuropsychol 2010 1 793 810

[B7] Piefke M Fink GR Recollections of one’s own past: The effects of aging and gender on the neural mechanisms of episodic autobiographical memory Anat Embryol (Berl) 2005 210 497 512 10.1007/s00429-005-0038-0 16172875

[B8] Onur ÖA Piefke M Lie C Thiel CM Fink GR Modulatory Effects of Levodopa on Cognitive Control in Young but not in Older Subjects: A Pharmacological fMRI Study J Cogn Neurosci 2011 23 2797 2810 10.1162/jocn.2011.21603 21254797

[B9] Chaytor N Schmitter-Edgecombe M Burr R Improving the ecological validity of executive functioning assessment Arch Clin Neuropsychol 2006 21 217 227 10.1016/j.acn.2005.12.002 16554143

[B10] Campbell Z Zakzanis KK Jovanovski D Joordens S Mraz R Graham SJ Utilizing virtual reality to improve the ecological validity of clinical neuropsychology: an FMRI case study elucidating the neural basis of planning by comparing the tower of London with a three-dimensional navigation task Appl Neuropsychol 2009 16 295 306 10.1080/09084280903297891 20183185

[B11] Parsons TD Silva TM Pair J Rizzo AA Westwood JD, Haluck RS, Hoffman HM, Mogel GT, Phillips R, Robb RA, Vosburgh KG Virtual environment for assessment of neurocognitive functioning: virtual reality cognitive performance assessment test Medicine meets virtual reality 16: parallel, combinatorial, convergent: NextMed by design 2008 Amsterdam: Ios Press 351 356

[B12] Rose FD Brooks BM Rizzo AA Virtual reality in brain damage rehabilitation: review Cyberpsychol Behav 2005 8 241 262 discussion 263–271 10.1089/cpb.2005.8.241 15971974

[B13] Weiss PL Kizony R Feintuch U Katz N Selzer ME, Clarke S, Cohen LG, Duncan PW, Gage FH Virtual reality in neurorehabilitation Textbook of neural repair. Volume II 2006 Cambridge: University Press 182 197 Medical Neurorehabilitation

[B14] Rizzo AA Schultheis MT Kerns KA Mateer CA Analysis of assets for virtual reality applications in neuropsychology Neuropsychol Rehabil 2004 14 207 239 10.1080/09602010343000183

[B15] Castelnuovo G Priore C Liccione D Cioffi G Virtual Reality based tools for the rehabilitation of cognitive and executive functions: the V-STORE PsychNology J 2003 1 310 325

[B16] Tarr MJ Warren WH Virtual reality in behavioral neuroscience and beyond Nat Neurosci 2002 5 Suppl 1089 1092 12403993 10.1038/nn948

[B17] Piefke M Onur ÖA Fink GR Aging related changes of neural mechanisms underlying visual-spatial working memory Neurobiol Aging 2012 33 7 1284 1297 10.1016/j.neurobiolaging.2010.10.014 21130531

[B18] Ponsford J Harrington H Olver J Roper M Evaluation of a community-based model of rehabilitation following traumatic brain injury Neuropsychol Rehabil 2006 16 315 328 10.1080/09602010500176534 16835154

[B19] Bennett TL Neuropsychological evaluation in rehabilitation planning and evaluation of functional skills Arch Clin Neuropsychol 2001 16 237 253 14590176

[B20] Cromby JJ Standen PJ Newman JP Tasker H Successful transfer to the real world of skills practised in a virtual environment by students with severe learning difficulties 1996 Maidenhead, UK: Proc 1st Euro Conf Disability, Virtual Reality & Assoc Tech 103 107

[B21] Parsons TD Neuropsychological Assessment Using Virtual Environments: Enhanced Assessment Technology for Improved Ecological Validity. Advanced Computational Intelligence Paradigms in Healthcare 6 Virtual Reality in Psychotherapy, Rehabilitation, and Assessment 2011 271 289

[B22] Lee J Ku J Cho W Hahn W Kim I Lee S Kang Y Kim D Yu T Wiederhold B A virtual reality system for the assessment and rehabilitation of the activities of daily living Cyberpsychol Behav 2003 6 383 388 10.1089/109493103322278763 14511450

[B23] Rand D Weiss PL Katz N Training multitasking in a virtual supermarket: a novel intervention after stroke Am J Occup Ther 2009 63 535 542 10.5014/ajot.63.5.535 19785252

[B24] Knight C Alderman N Burgess P Development of a simplified version of the multiple errands test for use in hospital settings Neuropsychol Rehabil 2002 12 231 255 10.1080/09602010244000039

[B25] Werner P Rabinowitz S Klinger E Korczyn A Josman N Use of the Virtual Action Planning Supermarket for the Diagnosis of Mild Cognitive Impairment Dement Geriatr Cogn Disord 2009 27 301 309 10.1159/000204915 19252401

[B26] Josman N Hof E Klinger E Marie RM Goldenberg K Weiss PL Kizony R Performance within a virtual supermarket and its relationship to executive functions in post-stroke patients. In Virtual Rehabilitation, 2006 International Workshop 2006 106 109

[B27] Klinger E Chemin I Lebreton S Marié R Virtual Action Planning in Parkinson’s Disease: AControl Study Cyberpsychol Behav 2006 9 342 347 10.1089/cpb.2006.9.342 16780402

[B28] Josman N Klinger E Kizony R Performance within the Virtual Action Planning Supermarket (VAP-S): An executive function profile of three different populations suffering from deficits in the central nervous system 2008 Maia, Portugal: Proc 7th ICDVRAT

[B29] Dyck E Schmidt H Botsch M OctaVis: A Simple and Efficient Multi-View Rendering System 2010 GI VR/AR Workshop 1 8

[B30] Wiederhold BK Wiederhold MD Lessons Learned From 600 Virtual Reality Sessions Cyberpsychol Behav 2000 3 393 400 10.1089/10949310050078841

[B31] Witmer BG Singer MJ Measuring Presence in Virtual Environments: A Presence Questionnaire Presence Teleop Virt 1998 7 225 240 10.1162/105474698565686

[B32] Lin JWJ Enhancement of user-experiences in immersive virtual environments that employ wide-field displays. University of Washington, Unpublished doctoral thesis Department of Industrial Engineering 2004

[B33] Lombard M Ditton TB Grabe ME Reich RD The role of screen size in viewer responses to television fare Comm Rep 1997 10 95 106 10.1080/08934219709367663

[B34] Elger CE Helmstaedter C Kurthen M Chronic epilepsy and cognition Lancet Neurol 2004 3 663 672 10.1016/S1474-4422(04)00906-8 15488459

[B35] Schefft BK Dulay MF Fargo JD Szaflarski JP Yeh HS Privitera MD The use of self-generation procedures facilitates verbal memory in individuals with seizure disorders Epilepsy Behav 2008 13 162 168 10.1016/j.yebeh.2008.01.012 18343201

[B36] Witmer BG Jerome CJ Singer MJ The Factor Structure of the Presence Questionnaire Presence Teleop Virt 2005 14 298 312 10.1162/105474605323384654

[B37] Osterrieth P Le test de copie d’une figure complexe; contribution à l’étude de la perception et de la mémoire Arch Psychol 1944 28 215 285

[B38] Berry DTR Allen RS Schmitt FA Rey-Osterrieth Complex Figure: Psychometric characteristics in a geriatric sample Clin Neuropsychol 1991 5 143 153 10.1080/13854049108403298

[B39] Park HS Shin YW Ha TH Shin MS Kim YY Lee YH Kwon JS Effect of cognitive training focusing on organizational strategies in patients with obsessive‒compulsive disorder Psychiatr Clin Neurosci 2006 60 718 726 10.1111/j.1440-1819.2006.01587.x 17109706

[B40] Shin MS Park SY Park SR Seol SH Kwon JS Clinical and empirical applications of the Rey-Osterrieth Complex Figure Test Nat Protocol 2006 1 892 899 10.1038/nprot.2006.115 17406322

[B41] Lezak MD Neuropsychological assessment 2004 4 Oxford: Oxford Univ. Press

[B42] Luzzi S Pesallaccia M Fabi K Muti M Viticchi G Provinciali L Piccirilli M Non-verbal memory measured by Rey–Osterrieth Complex Figure B: normative data Neurol Sci 2011 32 1081 1089 10.1007/s10072-011-0641-1 21630034

[B43] Hamby SL Wilkins JW Barry NS Organizational quality on the Rey-Osterrieth and Taylor Complex Figure Tests: A new scoring system Psychol Assess 1993 5 27

[B44] Binder LM Constructional strategies on complex figure drawings after unilateral brain damage J Clin Exp Neuropsychol 1982 4 51 58 10.1080/01688638208401116 7096586

[B45] Shin M Park S Kim M Lee Y Ha T Kwon J Deficits of Organizational Strategy and Visual Memory in Obsessive-Compulsive Disorder Neuropsychology 2004 18 665 15506834 10.1037/0894-4105.18.4.665

[B46] Markowitsch HJ Staniloiu A Amnesic disorders Lancet 2012 380 1424 1440 10.1016/S0140-6736(11)61304-4 22503117

[B47] Squire LR Declarative and nondeclarative memory: Multiple brain systems supporting learning and memory J Cogn Neurosci 1992 4 232 243 10.1162/jocn.1992.4.3.232 23964880

[B48] Tolman E Cognitive maps in rats and men Psychol Rev 1948 55 189 18870876 10.1037/h0061626

[B49] Tversky B Cognitive maps, cognitive collages, and spatial mental models Spatial Information Theory A Theoretical Basis for GIS 1993 14 24

[B50] Johns C Blake E Cognitive maps in virtual environments: Facilitation of learning through the use of innate spatial abilities 2001 ACM 125 129

[B51] Tong FH Marlin SG Frost BJ Cognitive map formation in a 3D visual virtual world 1995 IRIS/PRECARN

[B52] Brooks B Attree E Rose F Clifford B Leadbetter A The specificity of memory enhancement during interaction with a virtual environment Memory 1999 7 65 78 10.1080/741943713 10645373

[B53] Matheis RJ Schultheis MT Rizzo AA Burdea G, Thalmann D, Lewis JA Learning and memory in a virtual office environment Proceedings of the 2nd International Workshop in Virtual Rehabilitation 2003 Piscataway: Rutgers University 48 54

[B54] Mayer RE The promise of multimedia learning: using the same instructional design methods across different media Learn Instr 2003 13 125 139 10.1016/S0959-4752(02)00016-6

[B55] Collins HR Pictorial encoding and testing impact recognition memory 2008 Santa Barbara: University of California

[B56] Gellevij M Van Der Meij H De Jong T Pieters J Multimodal versus unimodal instruction in a complex learning context J Exp Educ 2002 70 215 239 10.1080/00220970209599507

[B57] Mayer RE Multimedia aids to problem-solving transfer Int J Educ Res 1999 31 611 623 10.1016/S0883-0355(99)00027-0

[B58] Ngiam J Khosla A Kim M Nam J Lee H Ng AY Multimodal Deep Learning 2010 In

[B59] Davis ET Scott K Pair J Hodges LF Oliverio J Can audio enhance visual perception and performance in a virtual environment 1999 Human Factors and Ergonomics Society 1197 1201

[B60] Dinh HQ Neff Walker CS Kobayashi A Hodges LF Evaluating the importance of multi-sensory input on memory and the sense of presence in virtual environments 1999 222

[B61] Calabrese P Markowitsch HJ Recovery of mnestic functions after hypoxic brain damage Int J Rehabil Health 1995 1 247 260 10.1007/BF02214643

[B62] Tulving E Markowitsch HJ Episodic and declarative memory: role of the hippocampus Hippocampus 1998 8 198 204 10.1002/(SICI)1098-1063(1998)8:3<198::AID-HIPO2>3.0.CO;2-G 9662134

[B63] Péruch P Vercher J Gauthier G Acquisition of spatial knowledge through visual exploration of simulated environments Ecol Psychol 1995 7 1 20 10.1207/s15326969eco0701_1

[B64] Hoffman HG Prothero J Wells MJ Groen J Virtual chess: Meaning enhances users’ sense of presence in virtual environments Int J Hum Comput Interact 1998 10 251 263 10.1207/s15327590ijhc1003_3

[B65] Lombard M Ditton T At the heart of it all: The concept of presence J Comput-Mediat Commun 1997 3 0–0

[B66] Lott A Bisson E Lajoie Y McComas J Sveistrup H The effect of two types of virtual reality on voluntary center of pressure displacement Cyberpsychol Behav 2003 6 477 485 10.1089/109493103769710505 14583123

[B67] Rand D Kizony R Weiss PL Virtual reality rehabilitation for all: Vivid GX versus Sony PlayStation II EyeToy 2004 Citeseer 87 94

[B68] Usoh M Catena E Arman S Slater M Using presence questionnaires in reality Presence Teleoperators Virt Environ 2000 9 497 503 10.1162/105474600566989

[B69] Biocca F Kim J Choi Y Visual touch in virtual environments: An exploratory study of presence, multimodal interfaces, and cross-modal sensory illusions Presence Teleoperators Virt Environ 2001 10 247 265

[B70] Péruch P Wilson PN Active versus passive learning and testing in a complex outside built environment Cogn Process 2004 5 218 227 10.1007/s10339-004-0027-x

[B71] Riva G Mantovani F Gaggioli A Presence and rehabilitation: toward second-generation virtual reality applications in neuropsychology J Neuroeng Rehabil 2004 1 9 10.1186/1743-0003-1-9 15679950 PMC546411

[B72] Lessiter J Freeman J Keogh E Davidoff J A cross-media presence questionnaire: The ITC-Sense of Presence Inventory Presence: Teleoperators Virt Environ 2001 10 282 297 10.1162/105474601300343612

[B73] Niemann H Sturm W Thöne-Otto A Willmes K CVLT–California verbal learning test–Deutsche adaptation 2008 Frankfurt: Pearson

[B74] Helmstaedter C Lendt M Lux S Verbaler Lern-und Merkfähigkeitstest. Beltz-Test 200122831704

[B75] Hoppe C Stojanovic J Elger CE Enhancing memory for lists by grouped presentation and rehearsal: a pilot study in healthy subjects with unexpected results Seizure 2009 18 711 715 10.1016/j.seizure.2009.10.001 19875311

[B76] LaViola JJ Jr A discussion of cybersickness in virtual environments ACM SIGCHI Bull 2000 32 47 56 10.1145/333329.333344

