# Supplementary material for: Learning real-life cognitive abilities in a novel 360°-virtual reality supermarket: a neuropsychological study of healthy participants and patients with epilepsy
Source: J Neuroeng Rehabil. 2013 Apr 23;10:42. doi: 10.1186/1743-0003-10-42 (PMC3637817; doi:10.1186/1743-0003-10-42)
Supplement: Additional file 1 — Clinical and demographic characteristics of the epilepsy patients subgroup. [file 1743-0003-10-42-S1.pdf]

Additional File 1 Clinical and demographic characteristics of the epilepsy patiens subgroup

ID	age	sex	type of epilepsy	lesion/ EEG	side of lesion
1	38	male	symptomatic focal	temporo-parietal gliosis	right
2	47	male	cryptogenic focal	hippocampal sclerosis (suspected)	right
3	25	male	symptomtic focal	temporo-parietal posttraumatic	right
4	35	male	cryptogenic focal	temporal epileptoform EEG	bilateral
5	32	female	symptomatic focal	occipital periventricular leukomalacia	bilateral left>right
