# Supplementary material for: Learning real-life cognitive abilities in a novel 360°-virtual reality supermarket: a neuropsychological study of healthy participants and patients with epilepsy
Source: J Neuroeng Rehabil. 2013 Apr 23;10:42. doi: 10.1186/1743-0003-10-42 (PMC3637817; doi:10.1186/1743-0003-10-42)
Supplement: Additional file 2 — Explorative usability questionnaire. The self-constructed questionnaire includes eight items. The first four items comprise the task-score; the last four items comprise the technique-score. Items are translated from the German version used in the study. [file 1743-0003-10-42-S2.pdf]

Task Score:

1. All in all, how well do you think you have fulfilled the task?

Very Easy			Average			Very Difficult

2. How important was it to you to execute the task especially well?

Very Important			Average			Very Important

3. All in all, how easy/difficult was the task for you?

Very Easy			Average			Very Difficult

4. How well did you feel during the task?

Not Well			Average			Very Well

Technique Score:

1. Did the edges of the monitor disturb you?

Not At All			Somewhat			Very Much

2. Did the gaps in between the monitors disturb you?

Not At All			Somewhat			Very Much

3. How easy/difficult was it for you to identify the products presented on the screen?

Very Easy			Average			Very Difficult

4. How easy/difficult has it been for you to operate the supermarket?

Very Easy			Average			Very Difficult
