# Supplementary figures and images for: Learning real-life cognitive abilities in a novel 360°-virtual reality supermarket: a neuropsychological study of healthy participants and patients with epilepsy
Source: J Neuroeng Rehabil. 2013 Apr 23;10:42. doi: 10.1186/1743-0003-10-42 (PMC3637817; doi:10.1186/1743-0003-10-42)

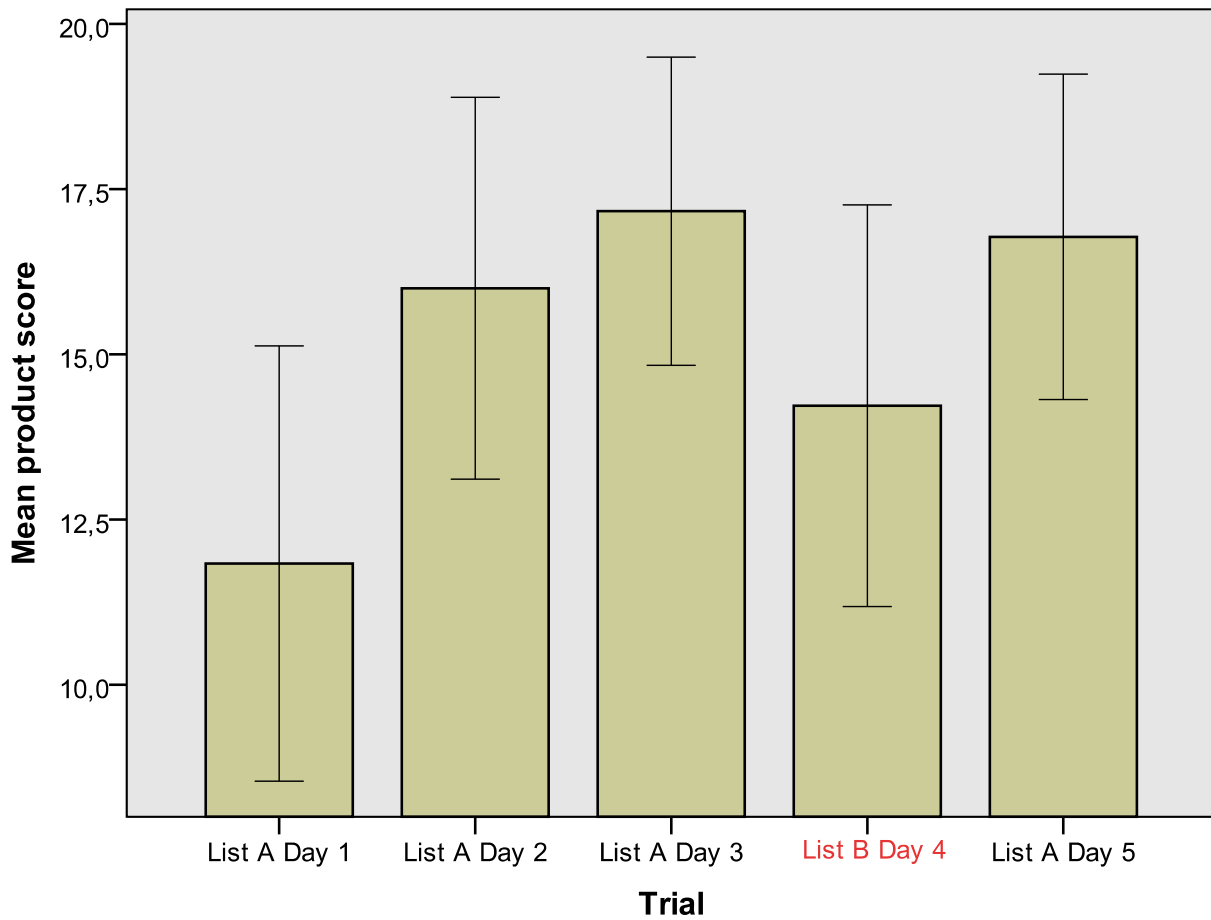

Supplement: Additional file 3 — Mean product score for the single trials of the five day VR program. Days 1 to 3 describe the product score (correct products minus false positives minus repetitions) for the consecutive learning of list A. Day 4 describes the product score for the interfering list B. Finally, day 5 describes the product score for the free recall of list A after the interference on day 4 (Note that on day 5, list A was not presented to the subjects again, but items should be recalled from the former learning trials). Error bars depict +/- 1 SD. [file 1743-0003-10-42-S3.pdf]
